# Pre- and Postsurgical Orthodontics in Patients with Moebius Syndrome

**DOI:** 10.1155/2017/1484065

**Published:** 2017-03-20

**Authors:** Marisabel Magnifico, Diana Cassi, Ilda Kasa, Marco Di Blasio, Alberto Di Blasio, Mauro Gandolfini

**Affiliations:** Section of Orthodontics, University Dental Center, Department of Biomedical, Biotechnological and Translational Sciences, University of Parma, Parma, Italy

## Abstract

The authors report a combined orthodontic-surgical correction of an adult patient's malocclusion affected by Moebius Syndrome (MS). The treatment was conducted at the Dentistry Unit and the Maxillofacial Surgery Unit of the University Hospital of Parma. Treatment of malocclusion was performed after the correction of facial mimic mobility with smile surgery. The postoperative stability and orthodontic results were good and the correction of the morphological problems related to the syndrome was very satisfactory.

## 1. Introduction

Moebius Syndrome is a rare disease characterized by permanent facial paralysis caused by decreased or absent formation of VI (abducens) and VII (facial) cranial nerve. In 1888, the ophthalmologist P. J. Moebius studied and classified the patients suffering from the concomitant congenital nonprogressive bilateral paralysis of these nerves. Since then, the eponymous Moebius Syndrome is used to indicate this condition [1].

MS is a rare syndrome with an incidence of one in every 100.000 live births with no gender predominance [2]. The disorder presents with varying phenotype and severity resulting in unilateral or bilateral paralysis of facial and abducens cranial nerve.

The etiology and pathogenesis of MS have not yet been clarified. Two main theories explain the disease to be due to an interruption in the vascular supply of the brainstem resulting in ischemia in the region of the facial cranial nerve nuclei owing to an environmental, mechanical, or a genetic cause [3–5] or an embryological developmental defect in the rhombomere segments including the facial cranial nerve nuclei [6, 7].

Mutations in the MBS1, MBS2, and MBS3 gene loci contribute to the development of MS through various pathways. HOX family genes coding for homeobox domains also have been implicated in MS [8, 9].

The clinical presentation of MS depends fundamentally on paralysis extension and structures involved. It is described as a close association between classic characteristics of the syndrome, such as paralysis of the facial nerve and the abducens nerve [10], and simultaneous involvement of other neural structures, mainly the II, V, IX, X, XI, and XII cranial nerve.

Other abnormalities include limbs malformations (clubfoot, absence or underdevelopment of the fingers, syndactyly, and brachydactyly), orofacial malformations (uvula bifida, cleft palate, underdevelopment of eyelid, epicanthal folds, hypertelorism, micrognathia, and deformity of the ear with hearing loss) [11], and other congenital syndromes such as Poland, Kallman, or Hanhart syndrome. Cardiovascular abnormalities are rarely present but can include dextrocardial, atrial, or ventricular septal defect, transposition of great vessels, and total anomalous pulmonary venous connection. Cranial nerve dysfunction often presents in infancy with difficulties such as inadequate sucking, necessitating nutritional supplementation, but in most cases inadequate sucking is treated with a special feeding bottle.

In cases suffering from a full expression of the syndrome, a significant association with various degrees of dental-skeletal deformities, in particular with micrognathia, was noted [12, 13]. Temporomandibular dysfunctions have also been described in MS. It is shown that correct mandibular motility is the key to its normal development, even in cases of growth cartilage loss [14]. The paralysis of the sixth and seventh cranial nerve and the involvement of other cranial nerves do not allow the establishment of a normal temporomandibular joint function leading to a reduction in the movements of maximum opening, protrusion, and laterality [15].

The knowledge and the therapeutic possibilities of alterations associated with the MS are significantly increased in recent decades; the rehabilitation protocols of strabismus and facial paralysis are now well known and well documented [16]. Free-muscle transplant innervated with motor nerves is currently the gold standard for facial animation in MS [17, 18]. In growing patients, this type of operation is performed at an early age to be followed latter by orthognathic surgery at complete maxillofacial growth [19]. Even in adult patients, facial animation should precede orthognathic surgery to prevent lip deformities and to ensure more predictable and satisfactory results [20].

In mild forms, in which there are residual muscle motor units involved in facial expressions, surgery can sometimes be replaced by speech therapy and physiotherapy, often with satisfactory results.

The absence of facial expression seriously affects the formation of patients' personalities. In fact, autism and other mental disorders were often overstated in the literature [21, 22]. Functional and aesthetics impairment of MS can also severely damage the emotional development of these young patients [23].

The classifications proposed over the years are different. The classification of Terzis and Noah is the one used at our facility [24]: this classification distinguishes cases based on the severity of the deficit, analyzed on clinical and instrumental (electromyography) basis, and divides them into the following:

- Moebius Syndrome: bilateral complete paralysis of the facial nerve and the abducens nerve
- Incomplete Moebius Syndrome: presence of residual movements in one side of the face
- Moebius-like forms: unilateral paralysis associated with the involvement of other cranial nerves

The treatment of these patients requires several specialists such as the pediatrician, speech therapist, neurologist, ophthalmologist, the psychiatrist, the geneticist, the maxillofacial surgeon, and the orthodontist [25–27].

## 2. Case Report

**Figure 1:**
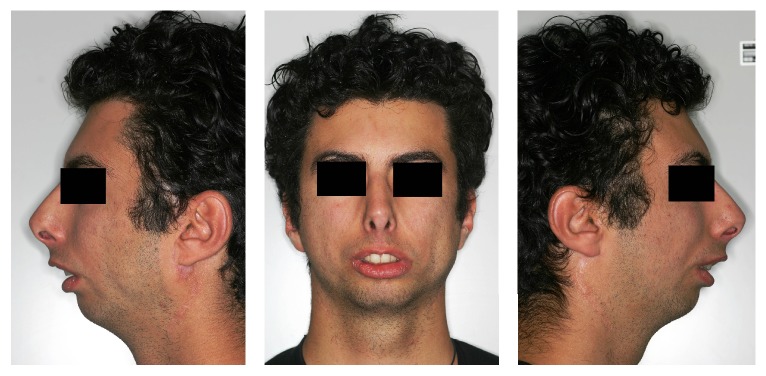
Extraoral pictures of profile before treatment.

A 23-year-old man came to our attention after he was diagnosed with MS by another institution. Family medical history was negative for use of drugs during pregnancy and for miscarriage threats, and no predisposing factors were found. Clinical examination showed a congenital bilateral complete palsy of facial nerve and dysfunction of lateral movements in both eyes, convex profile, reduced lower anterior facial height, open nasolabial angle, severe micrognathia, and incompetent lips with interlabial separation at rest of 13 mm (Figure 1).

**Figure 2:**
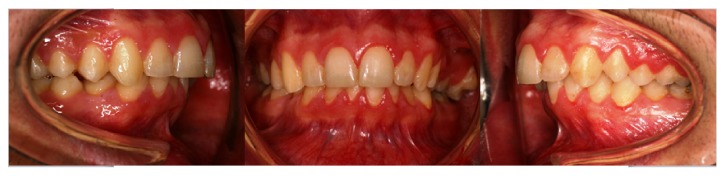
Intraoral occlusion before treatment.

Clinical intraoral examination before treatment showed a dental class II, division 1, with increased overjet (6 mm) and overbite (4 mm), retroinclination of upper incisors, II molar and canine class on both side, deviation of lower midline, complete dental formula, crowding in the lower jaw, and scissor bite of elements 2.7 and 2.8 (Figure 2).

Two years before orthognathic surgery, the patient has undergone smile surgery at Maxillofacial Surgery Unit of the University Hospital of Parma and consisted in double free muscle transfer using the gracilis muscle collected from the medial thigh and grafting it in the corners of the mouth and reinnervating it by the masseter motor nerve for facial animation. The elapsed time interval between the transplantation of the right side and that of the left side was six months.

**Figure 3:**
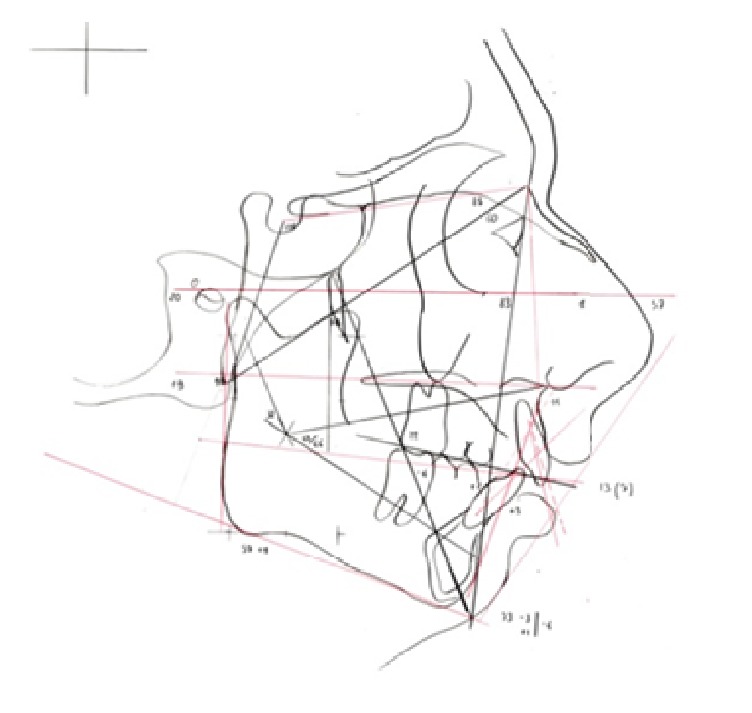
Cephalometric analysis.

The cephalometric analysis confirms the diagnosis of severe skeletal class II, retrognathic mandible, hypodivergent pattern and reduced lower facial height (Figure 3). The cephalometric analysis was conducted using as reference the Frankfurt plane, not the Natural Head Position [28], because we base assessments on Ricketts cephalometric analysis [29].

Considering the age of the patient, the severity of skeletal class II, and the vertical growth pattern, a combined surgical-orthodontic plan was formulated.

In accord with the surgeon, the following orthodontic treatment goals were set: solving crowding and correcting the lower midline and levelling and presurgical decompensation of the dental arches and the creation of a preoperative overjet sufficiently increased to support surgical mandibular advancement. In fact, the patient's initial occlusion showed a remarkable dental compensation to the skeletal pattern characterized by high proclination of the lower incisors and retroclination of the upper incisal group.

In the lower arch, the retraction and axis normalization of the incisors were achieved orthodontically by extractions of the lower first premolars and by maximum anchorage of the second premolars and the first molars. In the upper arch, a slight arch expansion was enough as the result of arch alignment for the tilt normalization of the maxillary incisors. The objectives of this presurgical orthodontic phase were indeed to get well-leveled and aligned dental arches with an overjet sufficiently large to support the next mandibular advancement.

Before orthodontic therapy, the patient has reached a stable periodontal situation and proper home hygiene maintaining by periodontal therapy and motivation.

**Figure 4:**
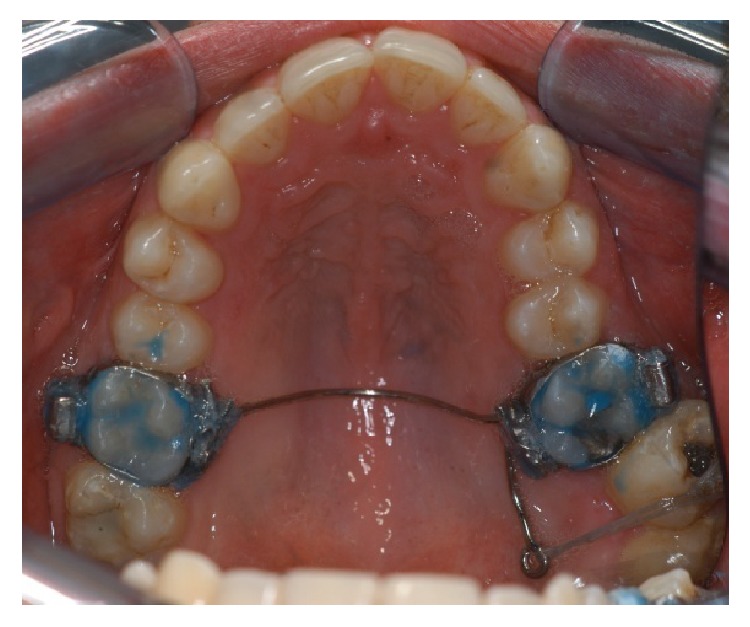
Palatal arch with extension arm.

The treatment begins with the correction of scissor bite of elements 2.7 and 2.8. A palatal arch was fixed on superior first molar, with an extension arm from 2.6 to the palatal side of 2.7. A button was positioned on the vestibular side of 2.7 and elastic ligation was tense from the button to the arm (Figure 4). Ligature was activated until the complete resolution of scissor bite.

**Figure 5:**
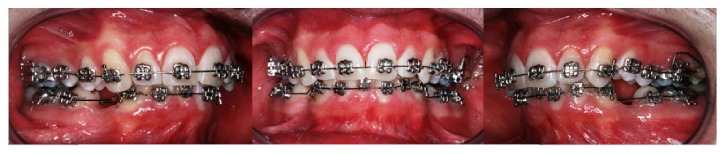
Extraction space before the closure.

In the upper arch the vestibular movement of the upper incisors was sufficient as a result of resolution of crowding to create a presurgical correct overjet (Figure 5).

**Figure 6:**
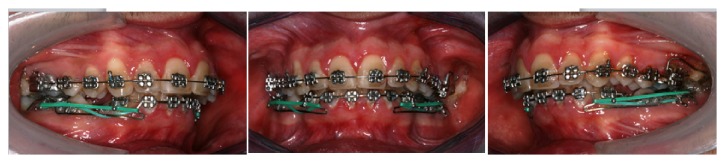
Segmented technique and intra-arch elastics to close extraction space.

In the lower arch, the anchorage for the retraction of the lower front group was obtained with the positioning of a Wilson® lingual arch. Ricketts' brackets with 0.018-inch slot were positioned on both dental arches [30]; the use of segmented technique and intra-arch elastics have allowed the closure of extraction spaces (Figure 6). Such segmented biomechanics was preferred to the use of miniscrews (TADs) for anatomic reasons and because complications, as failure or loss of miniscrew, are much more frequent in the lower arch [31].

**Figure 7:**
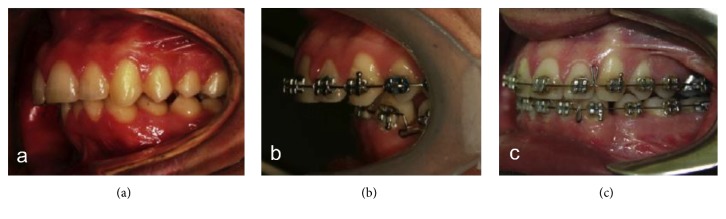
Lateral occlusion before orthodontic treatment (a), at the end of presurgical orthodontics (b), and after surgery (c).

Initial alignment of both dental arches was obtained using NiTi arch, 0.016 inches, followed by 0.016*∗*0.016-inch Elgiloy® and 0.016*∗*0.022-inch Elgiloy, also for levelling phase and for space closure in the lower arch and to obtain a positive torque in the upper front group (Figure 7).

**Figure 8:**
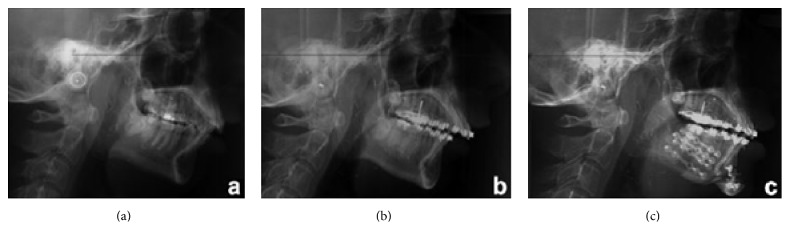
Teleradiography in lateral projection before orthodontic treatment (a), at the end of presurgical orthodontics (b), and after orthognathic surgery (c).

The patient so underwent surgery with mandibular osteotomy and advancement together with the correction of flat morphology of the chin obtained with genioplasty also to improve the total mandibular advancement. Upper lip incompetence that is typical in patients with MS has been corrected with lip augmentation through fat injections (Figure 8).

Orthodontics was continued after surgery to close minor spaces and to rehabilitate and restore the neuromuscular function and get final occlusal settling. Occlusal function was greatly improved using class II intermaxillary elastics. An individual positioner was used for retention for two years.

**Figure 9:**
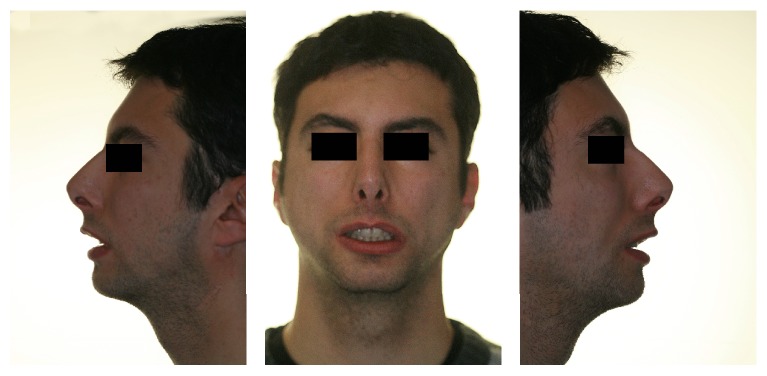
Profile after surgery.

**Figure 10:**
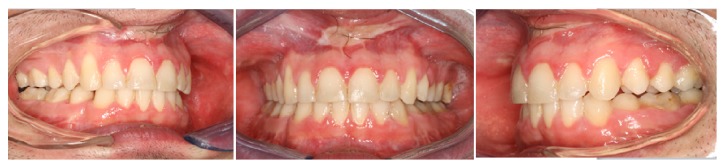
Occlusion after surgery.

The total orthodontic treatment duration was 24 months with 20 months of presurgical orthodontics and 4 months of postsurgical management. Outcome of the treatment was a significant improvement in the patient's smile and profile due to surgical normalization of skeletal pattern and smile surgery (Figure 9). Molar and canine relationships were corrected to class I with appropriate overjet and overbite and midlines were achieved (Figure 10).

## 3. Discussion

Moebius Syndrome is a complex disease with different manifestations that include paralysis of the VI and VII cranial nerve, dental abnormalities, limbs malformations, and other alterations. The expression of the syndrome shows considerable variability in signs and symptoms with different levels of severity and involvement of orofacial structures from isolated facial paralysis to severe craniofacial malformations.

Presence of facial paralysis is the most critical aspect in MS because it impedes expression of emotions in interpersonal relationships of these patients, leading to psychological problems due to difficulties in social interactions.

For these reasons, smile surgery should be performed as soon as possible also to reduce the psychological consequences of the syndrome to improve interpersonal relationships and psychophysical development of the patient.

Despite the presence of several works in literature on the MS, in particular on the treatment of facial paralysis and strabismus, the impact of dentoskeletal malocclusion on the syndrome is still poorly debated and underestimated. As already stated, micrognathia is frequently associated, and its surgical correction improves patient's function (oral competence, tooth protection, speech, and breathing) and aesthetics [20].

In cases of high severity dentofacial deformities, the smile surgery is not however sufficient to restore proper aesthetics and function; in these cases, a combined treatment is necessary. Moreover, in patients with MS in which smile surgery was performed after the osteotomy for mandibular advancement, abnormal stretching of the perioral tissues and worsening of the lower lip eversion with incisal exposure due to the absence of face muscle activity were observed [19].

Careful diagnosis and treatment planning combined with interdisciplinary discussions on planning the surgical aspects determine the success of these cases, particularly about the osteotomy plane, which must take account of previous treatment of gracilis transplantation. This is the best way to achieve stable, functional, and aesthetic results.

The surgical correction of such severe dentofacial deformities is a functional and esthetic surgery that affects patients' self-perception. The patient appreciated the improvement in his facial appearance after orthognathic surgery which was associated with a noted improvement in his psychosocial adjustments.

In some cases, the problems linked to the syndrome are such that it is still difficult to restore proper lip seal, and this can lead to a partial aesthetic defect related to residual labial incompetence.
